# Evaluation of Machine Learning Algorithms for Malware Detection

**DOI:** 10.3390/s23020946

**Published:** 2023-01-13

**Authors:** Muhammad Shoaib Akhtar, Tao Feng

**Affiliations:** School of Computer and Communication, Lanzhou University of Technology, Lanzhou 730050, China

**Keywords:** malware, cyberattacks, IoT, malicious threats, machine learning classifiers, RF, DT, cyber security, suspicious activity, SGD, extra trees, Gaussian NB

## Abstract

This research study mainly focused on the dynamic malware detection. Malware progressively changes, leading to the use of dynamic malware detection techniques in this research study. Each day brings a new influx of malicious software programmes that pose a threat to online safety by exploiting vulnerabilities in the Internet. The proliferation of harmful software has rendered manual heuristic examination of malware analysis ineffective. Automatic behaviour-based malware detection using machine learning algorithms is thus considered a game-changing innovation. Threats are automatically evaluated based on their behaviours in a simulated environment, and reports are created. These records are converted into sparse vector models for use in further machine learning efforts. Classifiers used to synthesise the results of this study included kNN, DT, RF, AdaBoost, SGD, extra trees and the Gaussian NB classifier. After reviewing the test and experimental data for all five classifiers, we found that the RF, SGD, extra trees and Gaussian NB Classifier all achieved a 100% accuracy in the test, as well as a perfect precision (1.00), a good recall (1.00), and a good f1-score (1.00). Therefore, it is reasonable to assume that the proof-of-concept employing autonomous behaviour-based malware analysis and machine learning methodologies might identify malware effectively and rapidly.

## 1. Introduction

Cyberattacks from hackers are currently the leading cause of concern in the technological world.

Traditional antivirus systems that rely on signature matching often overlook polymorphic and newly discovered hazardous executables; therefore, this is an issue that has to be explored, as well as the rapid spread of malware on the Internet. As they spread throughout the Internet and into new locations, viruses and other types of malware become more widespread and hazardous [1]. Static analysis based on human heuristic inspection is no longer considered practicable or efficient in light of the increasing growth of malware. However, several novel approaches to detecting and preventing malware are now in development. One strategy for malware detection recommends integrating data mining techniques, such as machine learning algorithms, with autonomous dynamic malware analysis [2].

**Figure 1:**
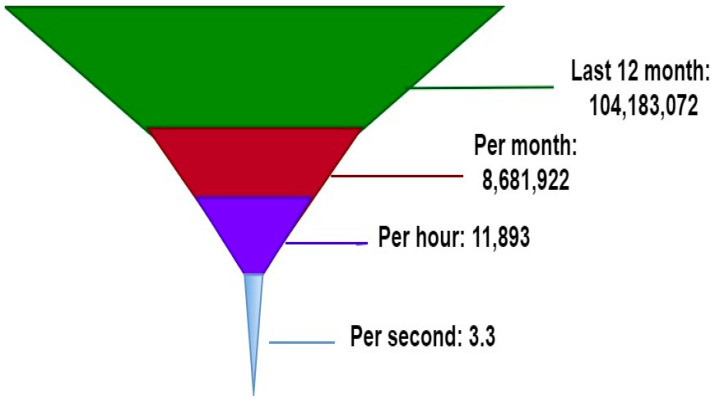
Threats of new malware per second.

These days, guarding computer networks is a top priority for security experts. Malware incidents have been on the rise despite the widespread availability of virus scanners and malware detection programmes. Dynamic and static approaches to malware detection and categorisation have also been proposed. There may be benefits of using a dynamic method to identify malware rather than a static method. This is due to the fact that, in contrast to static malware detection, hiding dangerous behaviour during execution is far more difficult [3,4]. In recent years, experts in the field of cybersecurity have been emphasising the use of machine learning algorithms for the purpose of detecting malware and predicting the behaviour of malware families. However, it does not appear that there is a consolidated repository that compares and rates various machine learning approaches to identifying fraudulent data. We conducted a battery of experiments to compare different machine learning strategies for identifying malware and classifying it into evolving family clusters. Threats of new malware per second is listed in Figure 1 [5].

**Figure 2:**
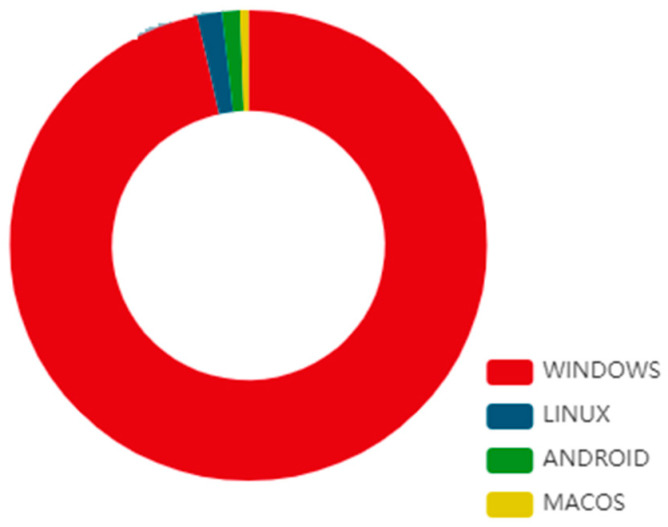
Classification of OS-based malware threats.

A collected dataset of authentic malware samples has been run by innocuous programmes from VirusTotal in a sandboxed setting to record malware behaviour, which we subsequently used to assess machine learning techniques in terms of commonly employed performance metrics [6,7]. Execution data collected as JSON reports provide us with a promising set of attributes defining the behaviour of a malware sample. When this is complete, the resultant feature set may be used to distinguish harmful from benign files. The motivation of this work came from the fact that several approaches have been created to optimize for a wide variety of criteria. As a result, they act differently, even when presented with the same circumstances. We also offer guidelines for researchers to follow and future research directions to take when tackling the challenge of dynamically recognising malware with machine learning techniques. The classification of OS-based threats is depicted in Figure 2 [8].

**Figure 3:**
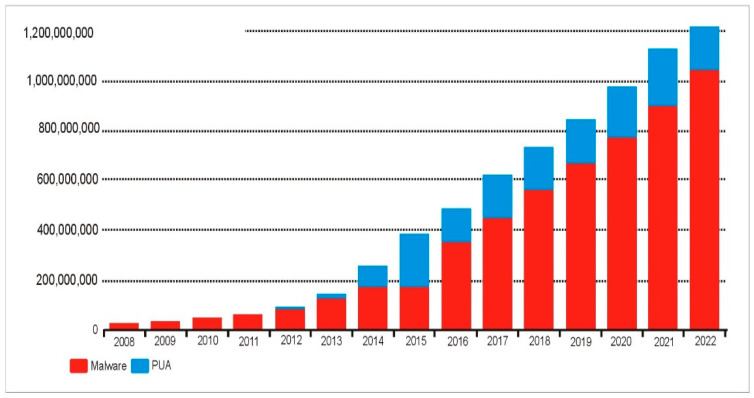
Total amounts of malware and potentially unwanted applications (PUAs).

More and more people are getting their information via the Internet through more and more different kinds of devices, from desktop PCs to embedded systems. Having more people have access to the Internet has been incredibly beneficial because of many advantages it provides, such as the possibility of rapid communication [9]. As long as there is the Internet connectivity, users may log in and use their preferred online services whenever they want. There is no surprise that malware has proliferated as the Internet use has increased, as it provides a lucrative market for illegal software makers. The exponential growth in malware detection seen in Figure 3 occurred only in the past few years [10,11,12].

Anti-malware, intrusion detection and other forms of detection have all emerged as responses to the harm caused by malware. However, there are certain urgent issues that require immediate attention, in light of ever-changing techniques employed by malicious software and the widespread presence of security flaws in popular programmes [13,14]. Many approaches from different areas have been presented for efficient malware detection. Since it is more difficult to disguise the destructive behaviour of malware while it is being run, dynamic techniques have proven more effective than static ones. As researchers have discovered the benefits of dynamic and automated techniques, they have shifted their focus away from traditional methods of malware detection [15].

## 2. Literature Review

**Figure 4:**
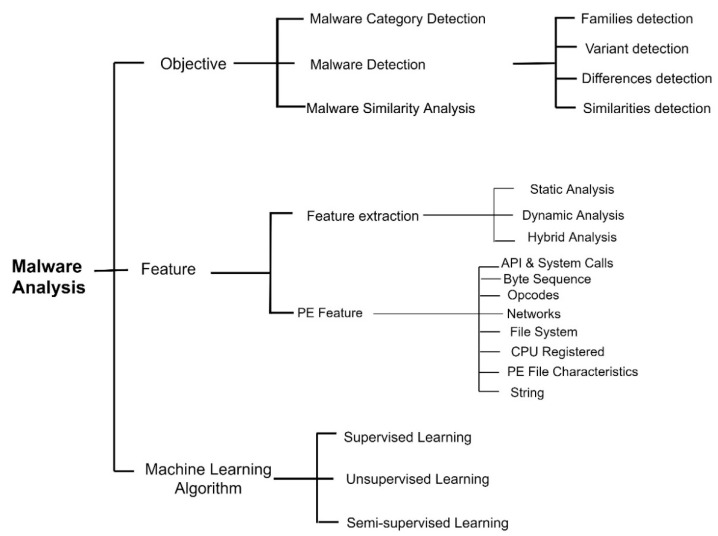
Malware analysis methods [17].

A novel representation for tracking the actions of malicious programmes, dubbed MIST, was proposed by Trinius (2016). The representation has been fine-tuned for the efficient study of behaviour using data mining and machine learning. During malware investigation, it can be automatically gathered using a behaviour-monitoring programme or manually converted from existing behaviour reports. Rieck (2018) attempts to use commonalities between malicious programmes to categorise them [16]. According to the Patil (2020), there are consistent patterns of behaviour among malware versions that can be used to infer the authors’ intentions. The first step in their process involves observing how malware behaves in a sandbox setting; the second relies on a corpus of malware annotated by an antivirus programme; and the third involves analysing the results in Figure 4 [17].

Learning methods are used to train a malware behaviour classifier, and the most distinguishing characteristics of the behaviour models are prioritised in order to provide an explanation for the classifications made. To analyse malware’s activity automatically, Rieck (2018) proposes a methodology based on machine learning [18]. The framework can classify unknown malware into previously identified classes based on their behaviours. Christodorescu (2018) proposes a method that uses a comparison between the execution patterns of known malware and a collection of innocuous apps to identify potential threats. The authors extract harmful features from a known virus that are absent in a collection of innocuous software. Malware detectors can use the results of the authors’ algorithm to identify new malware [19].

Machine learning algorithms concentrate on improving the quality of features by means of engineering, selection and representation. The model is trained with data representing the features of each class, producing a goodware/malware plane. This plane can be used to distinguish between malicious and legitimate software [20]. Understanding the domain is crucial for engineering and feature selection. Traditional machine learning-based malware detection systems have a problem, that is, they can be attacked if bad guys figure out how to reverse engineer, understand and represent the characteristics used by the model. Having a wide variety of examples to learn from is essential for machine learning algorithms. Privacy and security concerns mean that there is a scarcity of high-quality data that can be used for malware analysis in the public domain. Many researchers now create their own data sets for study using the same procedures developed by data scientists [21]. To examine Ye’s (2017) material would be an enormous task. All of these things make it difficult to develop a malware detection system that uses machine learning in real time [22]. 

Contemporary AI systems employ deep learning models, a refined version of the neural network model, to carry out a wide variety of tasks in natural language processing and robotics. It makes an effort to save a detailed representation of features in its hidden layers during training and can learn from its errors. Neelam (2020) examines studies that use deep learning models for malware analysis [23].

In 2015, Microsoft held a malware classification competition using the Kaggle platform. The provided database contained around 20,000 malware samples or almost half a terabyte. Using this information, Ronen (2015) analysed published studies and proposed studies in the field [24].

Souri (2020) did a thorough literature study of the various strategies given for malware detection using only data analysis techniques. The relevant scientific literature was partitioned into two groups, with one for each signature and behaviour type, and a comparative analysis of methods was carried out. In addition, recent studies have shown that hybrid techniques are more accurate than static or dynamic analysis alone; therefore, they should not be neglected [25].

Researcher Y. Yanfang (2018) summarised previous work on cloud-based malware detection methods, feature extraction and classification tactics and current malware development trends. This research also analysed and compared studies that used static analysis, dynamic analysis and a hybrid approach. However, the latest year covered by these analyses is 2017. The larger scale of recent investigations has made it clear that this initiative has to be expanded [26,27].

In a 2017 article, Ucci summed up various machine learning algorithms under consideration for the identification of malicious PE files in Windows OS. The studies were systematically arranged in accordance with many criteria, including the aims, methodology and sample characteristics of each individual study [28]. The economics of malware analysis, which we proposed to rename “malware analysis economy”, are discussed, along with the associated difficulties. Now, it has been three years, since the original study was published, and more research is warranted [29,30].

**A**. **Research Gap**

Cybercriminals create harmful software and introduce it into several computer systems in an effort to gain access or cause damage. Antivirus software, log file analysis and interaction monitoring are all used by businesses to look for telltale signs of malicious or suspicious behaviour that may indicate a recognised threat or attack trend [1]. Effective results may be obtained when using signature-based malware detection systems to identify well-known threats; nevertheless, these systems are easily bypassed by attackers. Increases in detection rates, decreases in false positive rates and reductions in processing time have been the subjects of much research into improving dangerous file detection. This sort of research is challenging to extend and develop due to a number of issues in the ecology of malicious software detection. In this study, we analysed many methods for finding malware in files that have already been released and discussed where more work needs to be performed. We took a look at the efforts being made to standardise the measurement, description, assessment and architecture enabling malware detection, and we pinpointed elements that may be valuable in making research on detecting harmful files more accessible and extensible [2].

## 3. Research Problem

The use of computers and their associated hardware is not only pervasive, but also intrinsically risky. This makes it possible for cybercriminals to create malicious software, take over computers and steal data [3]. Security professionals have a tough time providing constant, foolproof protection for computer systems because of a number of factors. In order to gain access or inflict damage, cybercriminals create malicious code and inject it into several computing systems. Organisations use antivirus software, log file analysis and interaction monitoring to identify patterns of behaviour that are consistent with known threats or attack vectors [4].

Malware’s harmful components might be uncovered via static analysis or dynamic analysis. Decompiling the virus and using a static analysis to parse malware files both aim to find harmful strings hidden inside the files. When the harmful code is run in a safe environment, such as a virtual computer, it may be monitored dynamically through analysis. Both methods have their advantages and disadvantages, but it is best to use both while analysing malware [5]. Better malware detection might be the result of less malicious features being employed in their development. That would give the researcher more time to go through the data. We are concerned that too many attributes are being used to detect malware when, in fact, a more limited collection of characteristics is needed. The initial step in deciding which malicious components to use is to identify possible approaches or algorithms [6]. There has to be a solution to the drastic decrease of both the amount of characteristics needed to detect malware and detect previously unseen malware [7,9]. 

## 4. Research Framework

**Figure 5:**
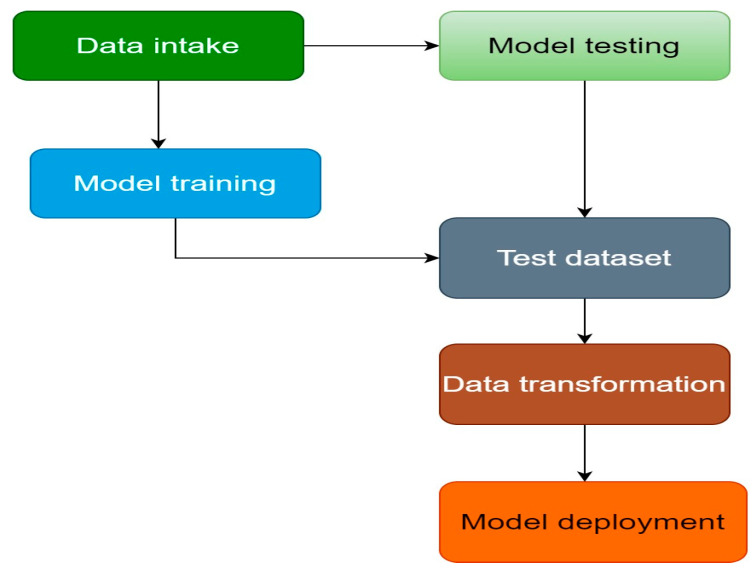
Research framework.

The proliferation of more complex kinds of malware poses a growing threat to modern computing infrastructure. Traditional signature-based malware detection technologies are becoming more useless due to the exponential proliferation of malware samples [10]. Machine learning has been proven by researchers to be able to accurately detect and label harmful files. Further, the accuracy of these machine learning models may be improved by using feature selection techniques to identify the most important features and reducing the size of the dataset, which results in fewer calculations. The research framework is depicted in Figure 5 [11].

**Figure 6:**
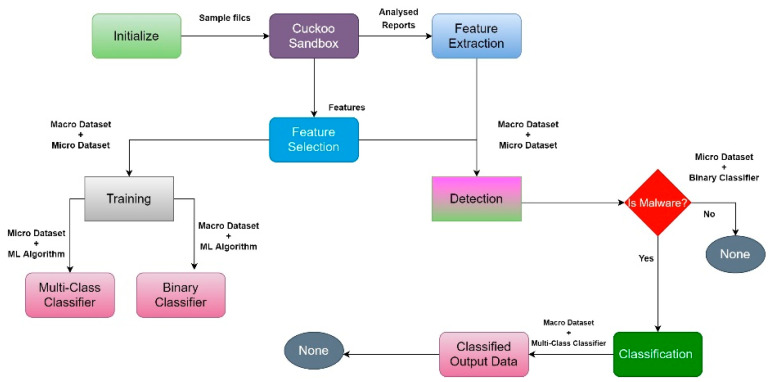
Malware detection framework structure.

In this study, we introduced a machine learning-based approach to malware analysis to enhance the efficiency and precision of malware detection and categorisation. We used the Cuckoo sandbox, which executes malware in an isolated environment and generates a report outlining its actions while in the sandbox to perform dynamic analysis [13,15,16]. In addition, we recommended a module for feature extraction and selection, which, as the name indicates, extracts features from the report before picking the most important qualities to ensure high accuracy with little computational cost. Then, for fine-grained classification and pinpoint detection, we employed a wide range of machine learning techniques. Our experimental results demonstrated higher detection and classification accuracy than state-of-the-art approaches. The malware detection framework structure is shown in Figure 6 [17].

## 5. Research Methodology

**Figure 7:**
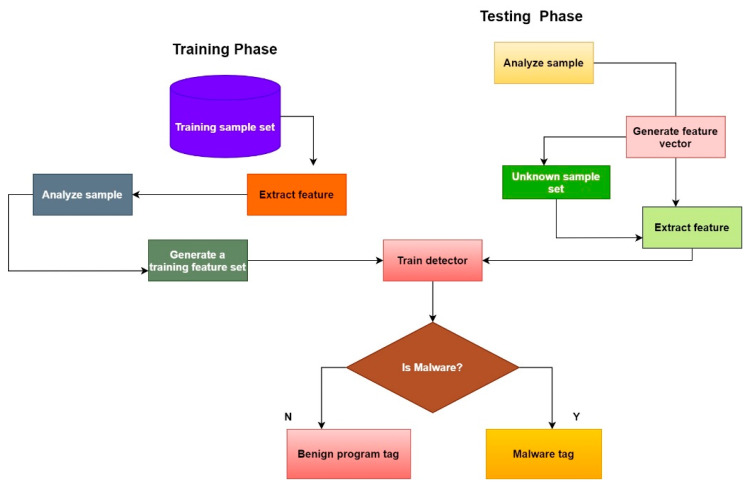
Proposed method of malware detection.

Figure 6 is a high-level overview of our machine learning-based malware detection procedure [18]. Some of the steps in this process include finding interesting datasets to train a classifier, detecting sophisticated malware and selecting features to include in the model. The following is a more in-depth explanation of the approach that was taken during this study. The proposed method is shown in Figure 7 [21].

### 5.1. DataSet

The selected dataset was taken from the Kaggle library. This training set was built by me using a combination of native and non-native characteristics extracted from Windows programs. There were 373 total samples in the file, of which 301 were malicious and the other 72 were safe. There were 531 characteristics listed, from F1 to F531, including a label column that indicates whether or not the file is harmful. The Kaggle data were used exclusively for the study. Many of the files in this archive included log data that were stolen by various forms of malware. A broad range of models may be trained using the recovered log information. It turned out that the samples were infected with malware from five different families. Included were more than 198,063 separate data points gathered from a wide variety of sources. There were 373 rows in the data and 531 columns as shown in Table 1 [22,23,24,25].

### 5.2. EDA and Visualisation

Features in the tens of thousands are common in modern datasets. The issue becomes more noticeable, as the number of characteristics in a machine learning model increases [26,27].

### 5.3. Features Selection

After new features are discovered through the process of feature extraction, the next step is to choose which features to use. Selecting features from a collection of newly recognised traits is called feature selection, and it is instrumental in improving the model accuracy, streamlining the model and reducing overfitting [28]. A variety of feature classification methods have been used by researchers to try and identify malicious software. As this study’s primary focus is on developing models to detect malware, the feature rank strategy is heavily utilised [29].

**Figure 8:**
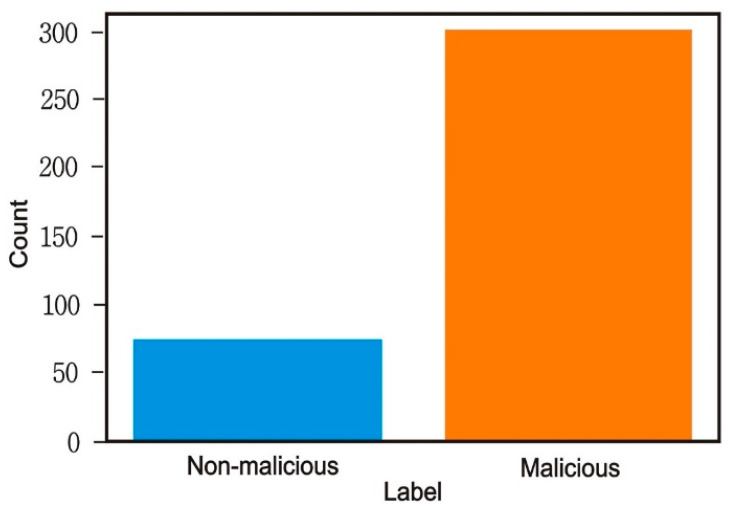
Counts of malicious and non-malicious data points after feature selection.

After features selections, from Figure 8, it is clear that our dataset data points weremore malicious which occupied 78% of all data points, and non-malicious data points occupied 22% of all data points.

## 6. Results and Discussion

For any categorisation technique to be effective, training and testing must be conducted. The system has to be trained with both potentially dangerous and benign data [1,7,15]. The use of machine learning techniques allows for the training of a classifier to automatically produce high-quality predictions. Classifiers such as the random forest, the SGD, extra trees and Gaussian neural networks all become better, when they are exposed to more and more labelled data. During the validation step, the classifier is presented with a set of new files, some of which are harmful and some of which are not, and asked to label them accordingly [18,19].

**Figure 9:**
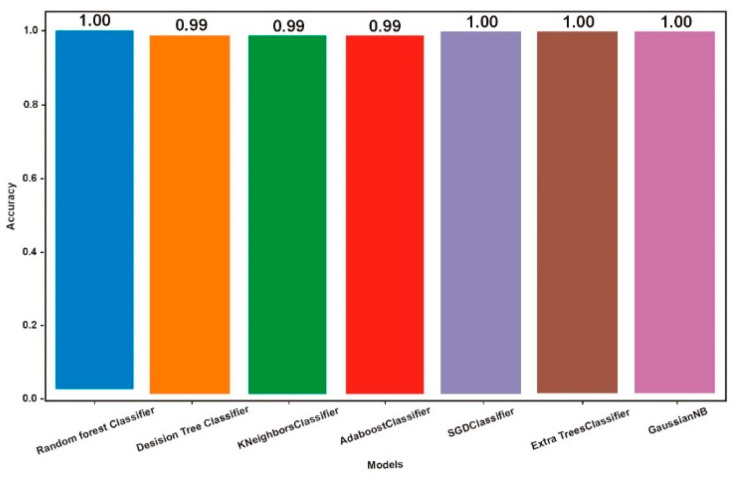
RF, SGD, extra trees and Gaussian NB Model.

A visual representation of the RF, the SGD, extra trees and Gaussian NB models is shown in Figure 9. A dropout is used in the final fully connected layer in the RF, SGD, extra trees and Gaussian NB models. In most cases, it appears that the dropout is used to simply add more layers to the model as a whole, rather than as a regularisation technique [21].

In this section, we reported the outcomes of an experimental evaluation of our suggested strategy for classifying and detecting malware. After generating a malware and cleanware dataset, it is put to use in testing. We analysed malware and placed it into different groups using a number of supervised machine learning methods, such as kNN, DT, RF, AdaBoost, SGD, extra trees and the Gaussian NB Classifier [22,25].

**Figure A1:**
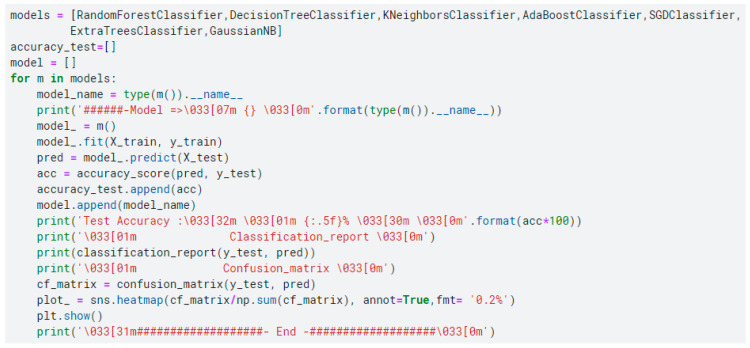
Testing of the models.

**Figure A2:**
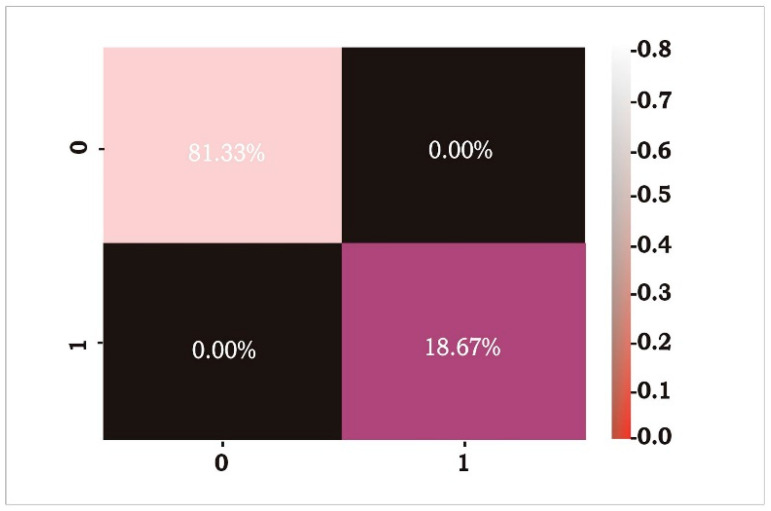
RF classifier model summary with a confusion matrix.

**Figure A3:**
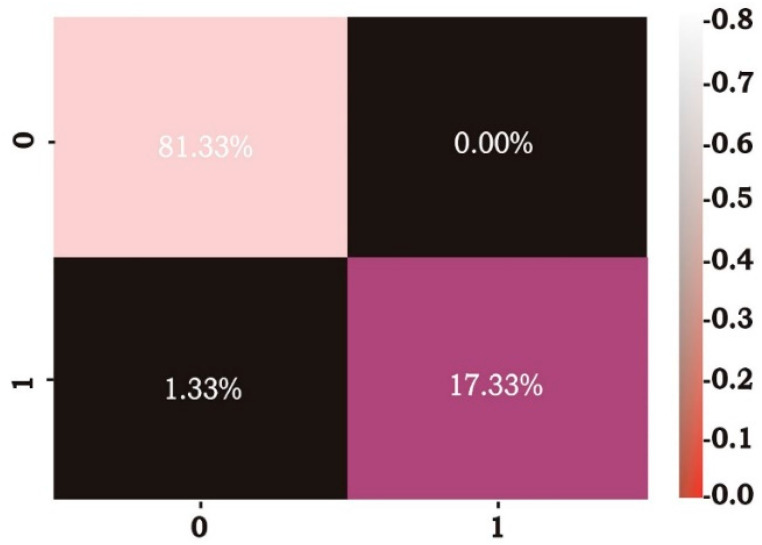
DT classifier model summary with a confusion matrix.

**Figure A4:**
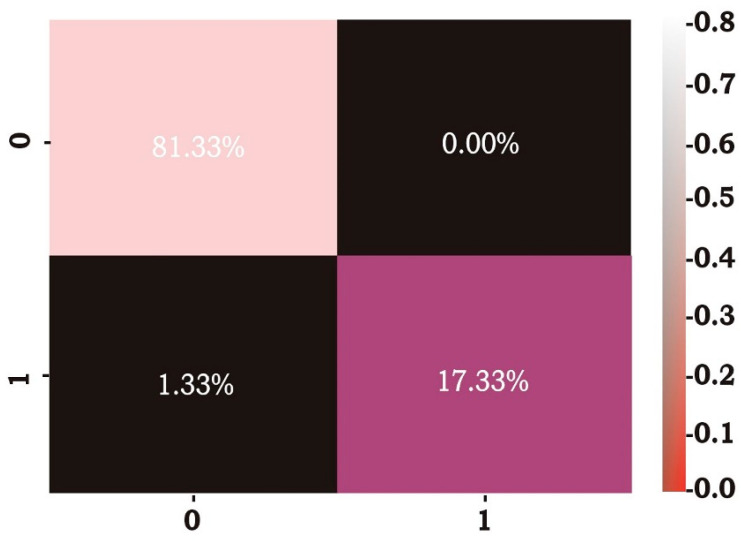
KNN classifier model summary with a confusion matrix.

**Figure A5:**
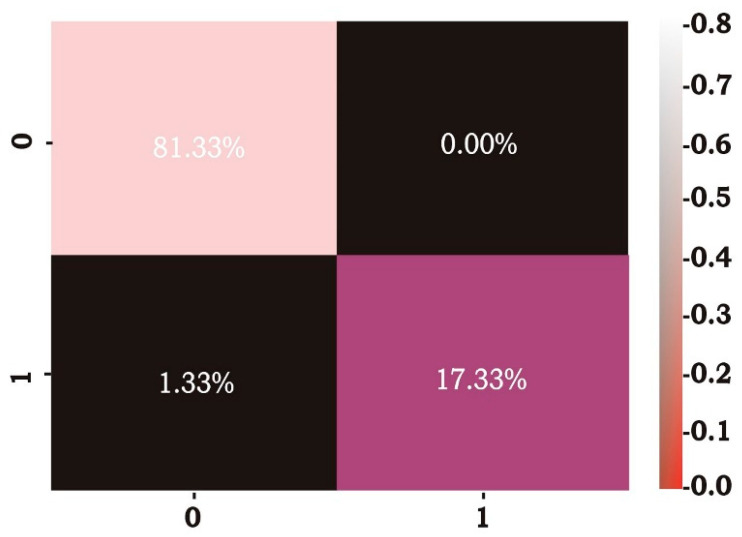
AdaBoost classifier model summary with a confusion matrix.

**Figure A6:**
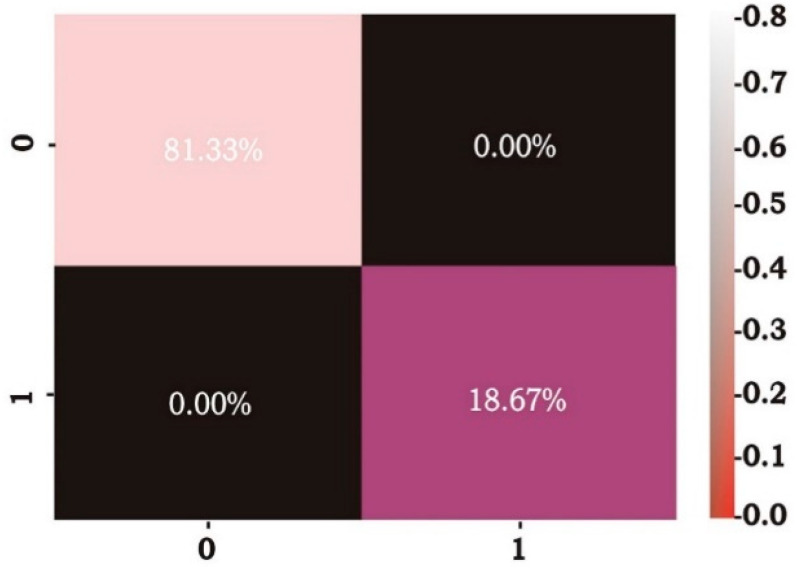
SGD classifier model summary with a confusion matrix.

**Figure A7:**
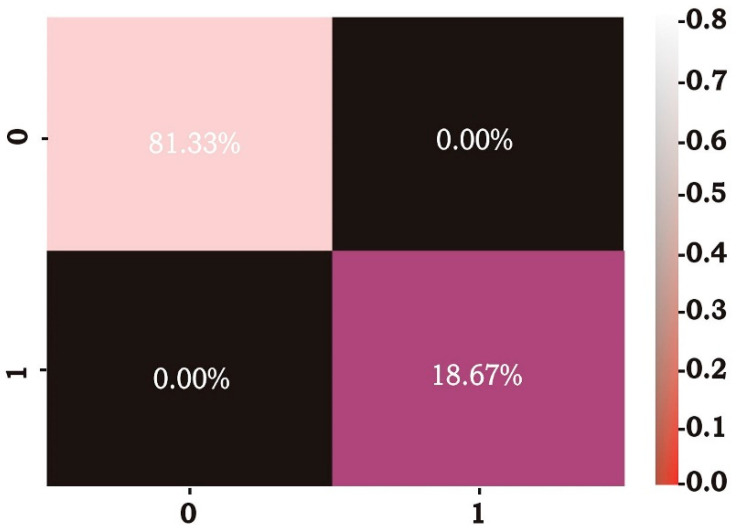
Extra Trees classifier model summary with a confusion matrix.

**Figure A8:**
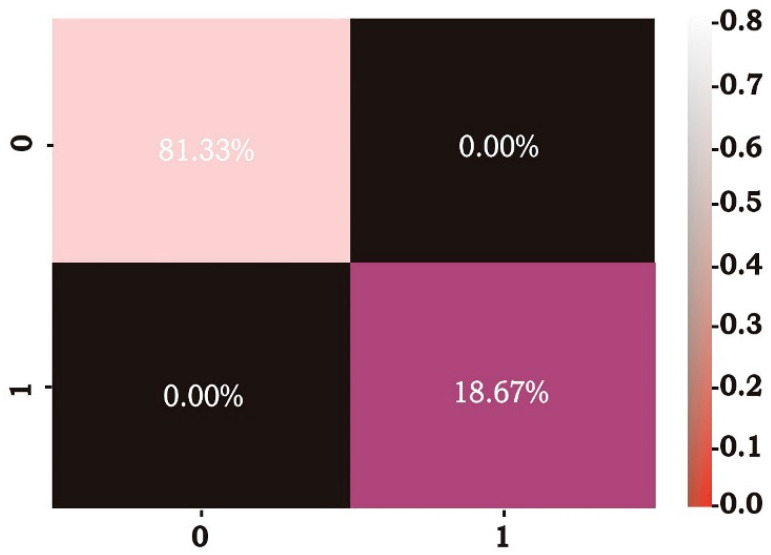
Gaussian NB classifier model summary with a confusion matrix.

Table 2 summarises the accuracies of the proposed Knn, RF, SGD, extra trees and Gaussian NB models. This study exemplified the growing interest in applying ML algorithmic approaches in malware detection across the academic community [26]. In this protection, we examined three machine learning methods for malware detection to see which one is most effective. In terms of detecting accuracy, the findings (Figure A1, Figure A2, Figure A3, Figure A4, Figure A5, Figure A6, Figure A7 and Figure A8, and Table A1, Table A2, Table A3, Table A4, Table A5, Table A6 and Table A7 in Appendix) demonstrated that the RF, SGD, extra trees and Gaussian NB models are the top classifiers, each having a perfect F1 score, a 100% accuracy, a 100% precision and a 100% recall [28].

Table 2 depicts that the RF, ASG, extra trees and Gaussian NB classifiers had a perfect F1 score, a 100% accuracy, a 100% precision and a 100% recall.

In terms of the online safety, malware is among the highest priorities. In reality, malware is the root of most Internet issues, including spam e-mails and DDoS attacks. That is, infected computers are frequently linked into larger networks called botnets, and many assaults are carried out by means of these hostile, attacker-controlled networks. In the fourth place, it discusses major problems and obstacles that researchers have to confront with. We focused in particular on the issue of idea drift and the difficulties of adversarial learning. It also analyses the issue of class imbalance and the current state of benchmarks used by the scientific community to measure the effectiveness of their approaches [29].

## 7. Conclusions

Finally, we concluded, to overcome the deficiencies of human feature construction and the limitations of existing learning approaches, this research layered the RF, ASG, extra trees and Gaussian NB models to create a novel ensemble deep neural network for malware detection. An F1 score of 100%, a precision of 100%, a recall of 100% and an accuracy of 100% were all achieved for the ASG, extra trees and Gaussian NB models. The proposed RF, ASG, extra trees and Gaussian NB models significantly improved the accuracy to detect malware, and the accuracy reached around 1 during training and very nearly 1 during testing. When we combine the RF, ASG, extra trees and Gaussian NB models, we are able to model sequence, learn from long-term dependency and extract spatially local correlations. To boost malware detection rates using ML algorithms, lower false positive rates and accelerate malware detection, several experts in the field have turned to machine learning approaches. Researchers divide data into a training set and a test set for a machine learning algorithm’s evaluation, with a training set used to teach the algorithm the desired function and a test set used to gauge how well the algorithm performs with the newly learned function.

## Figures and Tables

**Table 1:**
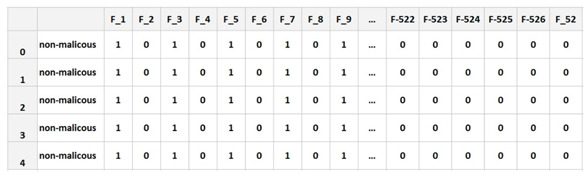
Dataset preview.

**Table 2:** Illustration of the accuracy.

	Model	Accuracy
**1**	RF	1.00
**2**	DT	0.99
**3**	KNN	0.99
**4**	AdaBoost	0.99
**5**	SGD	1.00
**6**	Extra Tree Classifier	1.00
**7**	Gaussian NB	1.00

## Data Availability

The data used to support the findings of this study are available from the corresponding author upon request.

## Appendix B

**Table A1:** RF classifier.

Classification Accuracy = 100%				
	Precision	Recall	F1-Score	Support
malicious	1.00	1.00	1.00	61.00
non-malicious	1.00	1.00	1.00	14.00
accuracy			1.00	75
macro avg	1.00	1.00	1.00	75
weighted avg	1.00	1.00	1.00	75

**Table A2:** DT classifier.

Classification Accuracy = 98.76%				
	Precision	Recall	F1-Score	Support
malicious	0.98	1.00	0.99	61.00
non-malicious	1.00	0.93	0.96	14.00
accuracy			0.99	75
macro avg	0.99	0.96	0.98	75
weighted avg	0.99	0.99	0.99	75

**Table A3:** KNN classifier.

Classification Accuracy = 98.69%				
	Precision	Recall	F1-Score	Support
malicious	0.98	1.00	0.99	61
non-malicious	1.00	0.93	0.96	14
accuracy			0.99	75
macro avg	0.99	0.96	0.98	75
weighted avg	0.99	0.99	0.99	75

**Table A4:** AdaBoost classifier.

Classification Accuracy = 98.71%				
	Precision	Recall	F1-Score	Support
malicious	0.98	1.00	0.99	61
non-malicious	1.00	0.93	0.96	14
accuracy			0.99	75
macro avg	0.99	0.96	0.98	75
weighted avg	0.99	0.99	0.99	75

**Table A5:** SGD classifier.

Classification Accuracy = 100%				
	Precision	Recall	F1-Score	Support
malicious	1.00	1.00	1.00	61
non-malicious	1.00	1.00	1.00	14
accuracy			1.00	75
macro avg	1.00	1.00	1.00	75
weighted avg	1.00	1.00	1.00	75

**Table A6:** Extra trees classifier.

Classification Accuracy = 100%				
	Precision	Recall	F1-Score	Support
malicious	1.00	1.00	1.00	61
non-malicious	1.00	1.00	1.00	14
accuracy			1.00	75
macro avg	1.00	1.00	1.00	75
weighted avg	1.00	1.00	1.00	75

**Table A7:** Gaussian NB classifier.

Classification Accuracy = 100%				
	Precision	Recall	F1-Score	Support
malicious	1.00	1.00	1.00	61
non-malicious	1.00	1.00	1.00	14
accuracy			1.00	75
macro avg	1.00	1.00	1.00	75
weighted avg	1.00	1.00	1.00	75
